# Targeting CYP4A attenuates hepatic steatosis in a novel multicellular organotypic liver model

**DOI:** 10.1186/s13036-019-0198-8

**Published:** 2019-08-08

**Authors:** Jae-Sung Ryu, Minji Lee, Seon Ju Mun, Sin-Hyoung Hong, Ho-Joon Lee, Hyo-Suk Ahn, Kyung-Sook Chung, Gun-Hwa Kim, Myung Jin Son

**Affiliations:** 1 Stem Cell Convergence Research Center, Korea Research Institute of Bioscience and Biotechnology (KRIBB), 125 Gwahak-ro, Yuseong-gu, Daejeon, 34141 Republic of Korea; 2 Drug and Disease Target Team, Division of Bioconvergence Analysis, Korea Basic Science Institute, Cheongju, Chungcheong 28119 Republic of Korea; 3 Department of Bio-Analytical Science, University of Science and Technology (UST), Daejeon, 34113 Republic of Korea; 4 Department of Functional Genomics, Korea University of Science & Technology (UST), 217 Gajungro, Yuseong-gu, Daejeon, 34113 Republic of Korea; 5 Biomedical Translational Research Center, KRIBB, 125 Gwahak-ro, Yuseong-gu, Daejeon, 34141 Republic of Korea; 6 Graduate School of Analytical Science and Technology (GRAST), Chungnam National University, Daejeon, 34134 Republic of Korea

**Keywords:** Liver, Hepatic steatosis, 3D, CYP4A, HET0016

## Abstract

**Background:**

Non-alcoholic fatty liver disease (NAFLD) begins as simple hepatic steatosis, but further progress to chronic liver diseases results in severe liver damage and hepatic failure. However, therapeutic options are scarce due to the lack of reliable human in vitro liver models for understanding disease progression mechanisms and developing therapies.

**Results:**

We describe here a novel method for generating 3D hepatic spheroids using HepaRG cells, vascular endothelial cells, and mesenchymal stem cells cultured on a thick layer of soft matrix in a narrow conical tube; this method improved self-organization efficiency and functional competence. We further developed a 3D hepatic steatosis model with excess glucose and palmitate, accurately recapitulating steatosis phenotypes such as neutral lipid accumulation, enhanced expression of lipogenesis and gluconeogenesis markers, increased intracellular triglyceride content, and reduced glucose uptake. The expression and activity of cytochrome P450 4A (CYP4A), a hepatic glucose and lipid homeostasis enzyme, that is highly expressed in liver tissues from NAFLD patients, was induced in our in vitro steatosis model, and inhibiting CYP4A with the selective inhibitor HET0016 or a specific siRNA ameliorated steatosis-related pathology through reduced ER stress and improved insulin signaling.

**Conclusions:**

We provide here a novel 3D human cell-based hepatic model that can be easily generated and reliably simulate hepatic steatosis pathology. We have experimentally validated its potential for target validation and drug evaluation by focusing on CYP4A, which may serve as a translational platform for drug development.

**Electronic supplementary material:**

The online version of this article (10.1186/s13036-019-0198-8) contains supplementary material, which is available to authorized users.

## Background

Hepatic steatosis, which is lipid accumulation in the liver due to an imbalance of lipid metabolism, is generally found in healthy populations and is known to be related to obesity and metabolic diseases such as type 2 diabetes mellitus (T2DM) [1–3]. Non-alcoholic fatty liver disease (NAFLD) starts with hepatic steatosis and can progress to chronic liver diseases, including non-alcoholic steatohepatitis (NASH, hepatic steatosis with inflammation), fibrosis, and cirrhosis [4, 5], and is ultimately associated with the incidence of hepatocellular carcinoma [6]. Simple lipid accumulation is considered one of the first steps in this chronic process that is characterized by accelerating histological damage caused by oxidative stress, inflammation, necrosis, and fibrosis [4, 7, 8]. Therefore, targeting steatosis in an early stage is important before the disease progresses to severe and irreversible stages.

Although various mouse models of NAFLD, including dietary, chemical, and genetic models, are used in preclinical drug development [9], there is a need for human cell-based liver models that would provide additional and more refined alternatives to animal experiments and, more importantly, resolve the issue of low predictive value due to interspecies differences. In particular, a hepatic model predicting human responses for drug evaluation is urgently needed, and patient-derived primary human hepatocytes (PHHs) are the ideal model for testing drug targets. However, the application of PHHs is limited by their low availability, difficult isolation procedure, and loss of proliferative capacity and long-term functionality in vitro [10]. HepaRG cells, a liver progenitor cell line that is widely used as an alternative and sustainable cell source for hepatocytes, proliferate and exhibit long-term phenotypical and functional stability after differentiation [11–13]. Recent advances in three-dimensional (3D) culture technologies have allowed investigators to recapitulate native liver functions through providing physiological environments, such as cell-to-cell or cell-to-extracellular matrix interactions [14–16]. The use of 3D HepaRG models for toxicity screening and fibrosis modelling has been evaluated [17–19]. However, 3D disease modelling, such as modelling of hepatic steatosis using HepaRG cells, has not been reported. Here, we provide a novel method to efficiently generate a multicellular 3D organotypic human hepatic steatosis model and further validate the feasibility of this platform for mechanism studies and for the validation of drugs targeting hepatic steatosis.

We previously identified 54 cytochrome P450 (CYP450) proteins that were upregulated in *db*/*db* and high-fat diet (HFD) diabetic mouse livers compared with normal mice and found that the CYP4a family, which includes important enzymes in lipid homeostasis that catalyze omega-hydroxylation of endogenous fatty acids and prostaglandin [20], was the most enriched isoform [21]. We found that inhibition of CYP4a by treatment with the specific inhibitor HET0016 [22] ameliorated severe hepatic steatosis in the diabetic mouse liver [21]. In addition, an increase in CYP4a expression has been reported in *ob*/*ob* mice [23] and in diet-induced mouse models of NASH [24, 25]. More importantly, potent expression of CYP4A was detected in liver tissues from NAFLD patients, and *CYP4a14−/−* mice were resistant to hepatic steatosis and fibrosis [25]. Therefore, unravelling the roles of CYP4A in the pathogenesis of hepatic steatosis and developing methods to target CYP4A may be a valuable approach to treating human hepatic steatosis.

In this study, we established a novel method to develop a functionally mature multicellular 3D organotypic liver model. To recapitulate the human disease phenotypes, we administered excess glucose and palmitate, the most abundant fatty acid in the human steatotic liver [26], during the generation of the hepatic organoid-like structure. We demonstrated the potential of our novel 3D human cell-based hepatic steatosis model as a versatile and valuable platform for target validation and drug evaluation through a study of CYP4A and its inhibitor.

## Results

### A novel method for spontaneous self-organization of 3D hepatic spheroids

**Fig. 1:**
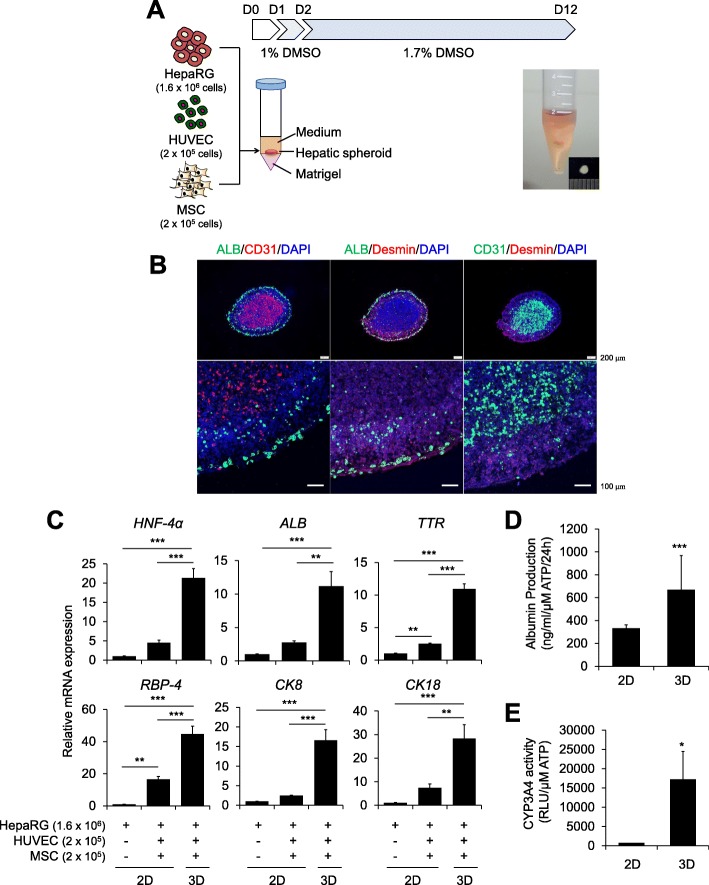
Generation of a novel multicellular organotypic liver model. **a** Schematic diagram of hepatic spheroid generation and macroscopic images of 3D spheroids. The indicated numbers of HepaRG cells, HUVECs, and MSCs were self-assembled on a Matrigel bed in a conical tube. **b** Distributions of HepaRG cells (ALB), HUVECs (CD31), and MSCs (Desmin) within cryosections of 3D hepatic spheroids on day 12. The nuclei were counter-stained with DAPI (blue). **c** mRNA expression levels of the indicated hepatocyte-specific markers were determined in differentiated HepaRG cells cultured alone or with HUVECs and MSCs in 2D or 3D triple co-culture using real-time PCR on day 12. β-Actin expression was used as an internal control; *n* = 3. **d** ALB production and **e** CYP3A4 activity were determined in 2D triple co-culture or 3D hepatic spheroids on day 12, normalized to the ATP concentration; *n* = 3. *p < 0.05, **p < 0.01, and ***p < 0.001

A self-organization protocol had been previously described in which hepatocytes derived from primary human tissue [27] or from pluripotent stem cells [28] were co-assembled with supporting cells on a thick layer of a soft matrix, such as Matrigel, in a 24-well plate. To generate a tissue-like organotypic 3D liver model, we also adapted a self-organization method using HepaRG cells, human umbilical vein endothelial cells (HUVECs), and mesenchymal stem cells (MSCs) on a Matrigel bed in a 24-well plate (Additional file 1: Figure S1A). However, we failed to generate distinct 3D cell clusters in over 30% of wells in a 24-well plate. Thus, we modified the culture format from a 24-well plate to a conical tube to provide a small and narrow area in which the cells could easily assemble, which yielded a 100% success rate for cluster generation. Next, we optimized the time schedule for the differentiation and 3D culture of HepaRG cells and the cell numbers for triple co-culture (Additional file 1: Figure S1B). When HepaRG cells were differentiated with 1.7% DMSO for 2 weeks after self-organization of the cells on Matrigel in a conical tube (Additional file 1: Figure S1B, protocol b), expression of the mature hepatocyte markers *Albumin* (*ALB)* and *TTR* was significantly increased compared with that in protocol a; self-organization was performed 1 week after 2D differentiation (Additional file 1: Figure S1B and C). When more HepaRG cells were used (1.6 × 10^6^ cells/spheroid), expression of the mature hepatocyte markers *ALB*, *CK8*, and *CYP3A4* was highest (Additional file 1: Figure S1B and D, protocol b3). When the differentiation period was shortened from 14 days to 10 days, the expression of the mature hepatocyte markers was not changed and even slightly increased in the case of *ALB* (Additional file 1: Figure S1B and E). Therefore, we used an 8:1:1 ratio of HepaRG cells:HUVECs:MSCs for self-organization and then differentiated the HepaRG cells in the presence of 1.7% DMSO for 10 days on Matrigel in a conical tube (Fig. 1a).

In the 3D hepatic spheroids, ALB-positive HepaRG cells and CD31-positive HUVECs were mainly localized in the peripheral and central regions of the spheroid, respectively, and Desmin-positive MSCs were dispersed, but were mainly located between the HepaRG cells and the HUVEs, consistent with previous data from our group [29] and others [30] (Fig. 1b). The expression of the mature hepatocyte markers *HNF-4α, ALB, TTR, RBP-4, CK8,* and *CK18* was highly increased in the 3D hepatic spheroids compared with that in either 2D culture of HepaRG cells alone or triple co-culture (Fig. 1c). More importantly, the production of ALB (Fig. 1d) and the activity of CYP3A4 (Fig. 1e), a main drug-metabolizing hepatic enzyme, were increased in 3D hepatic spheroids compared with that in 2D co-culture. Thus, we provide a novel, easy, and efficient method for the spontaneous self-organization of 3D hepatic spheroids with enhanced functionality as a multicellular organotypic liver model.

### Generation of a novel 3D hepatic steatosis model and increases in CYP4A and ER stress in a steatosis model

**Fig. 2:**
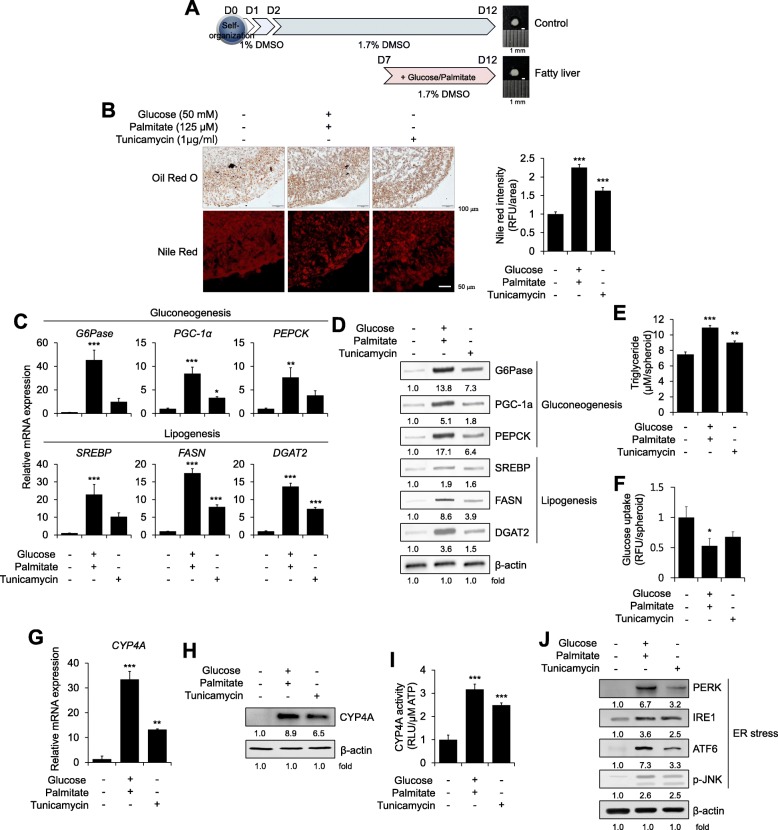
Generation of a novel 3D hepatic steatosis model and increases in CYP4A and ER stress in a steatosis model. **a** Brief outline of the generation of a 3D hepatic steatosis model after culture with high glucose and palmitate for 5 days. **b** Intracellular lipids were stained with Oil red O (top) and Nile red (bottom) and quantified by Nile red fluorescence intensity (right); *n* = 3. **c** mRNA and **d** Protein expression levels of gluconeogenesis- and lipogenesis-related genes were determined in control or glucose/palmitate, or tunicamycin (ER stressor)-treated 3D spheroids. β-Actin was used as an internal control for mRNA and protein expression; *n* = 3. **e** The triglyceride concentration was quantified by absorbance in each group; *n* = 3. **f** Glucose uptake was quantified by fluorescence intensity in each group; *n* = 3. **g** mRNA expression levels, **h** Protein expression levels, and **i** Activity of CYP4A were determined in control, glucose/palmitate-treated, or tunicamycin-treated 3D spheroids; *n* = 3. **j** Western blot analysis of ER stress markers in each condition. β-Actin was used as an internal control. *p < 0.05, **p < 0.01, and ***p < 0.001

Next, we generated a 3D hepatic steatosis model in which excess glucose and palmitate were added for 5 days during the later stage of self-organization (Fig. 2a). To optimize treatment conditions, we evaluated HepG2 cells in 2D culture, and treatment with 50 mM glucose and 125 μM palmitate significantly increased neutral lipid staining with Nile red (Additional file 1: Figure S2A) without affecting cell viability (Additional file 1: Figure S2B). Enhanced expression of the lipogenesis markers *SREBP* and *FASN* was determined under high-glucose and palmitate treatment conditions (Additional file 1: Figure S2C). Therefore, hepatic spheroids were treated with 50 mM glucose and 125 μM palmitate to recapitulate steatosis pathology, which induced high contents of total and neutral lipid storage droplets stained with Oil red O and Nile red, respectively (Fig. 2b). The expression levels of the gluconeogenesis markers *G6pase*, *PGC-1α*, and *PEPCK* were increased 45.3-, 8.44-, and 7.65-fold, respectively, and the lipogenesis markers *SREBP1*, *FASN*, and *DGAT2* were increased 22.8-, 17.5-, and 13.7 -fold, respectively, with high-glucose and palmitate treatment, respectively, compared with control (Fig. 2c). Consistent with these mRNA results, protein levels were also elevated in response to high-glucose and palmitate treatment (Fig. 2d). Quantitatively, the intracellular triglyceride content was increased by 1.46 ± 0.04-fold in steatosis-induced spheroids compared with that in the untreated control (Fig. 2e), and glucose uptake was reduced by 0.59 ± 0.07-fold under steatosis-inducing conditions (Fig. 2f). Therefore, these data reveal that high-glucose- and palmitate-treated self-organized 3D hepatic spheroids can exhibit steatosis phenotypes. Next, we verified the function of CYP4A and its value for targeting hepatic steatosis in our novel 3D multicellular organotypic liver model because high levels of CYP4A, an important enzyme in hepatic glucose and lipid homeostasis, were observed in liver tissues from NAFLD patients [25].

In our 3D steatosis model, CYP4A expression was induced by high-glucose and palmitate treatment at the mRNA (Fig. 2g) and protein (Fig. 2h) levels. CYP4A activity was also increased up to 3.16 ± 0.23-fold in high-glucose- and palmitate-treated spheroids compared with that in untreated controls (Fig. 2i). Under these conditions, the ER stress markers, such as protein kinase double-stranded RNA-dependent-like ER kinase (PERK), inositol-requiring enzyme 1 (IRE1), activating transcription factor 6 (ATF6), and phospho- c-jun N-terminal kinase (p-JNK) were increased by 6.7-, 3.6-, 7.3-, and 2.6-fold, respectively, compared with control (Fig. 2j). When ER stress was directly induced by treatment with tunicamycin, lipid droplets (Fig. 2b), expression of gluconeogenesis and lipogenesis markers (Fig. 2c and d), triglyceride content (Fig. 2e), and CYP4A expression (Fig. 2g and h) and activity (Fig. 2i) were also obviously promoted. These results demonstrate that ER stress induced by metabolic stress, such as high glucose and palmitate, is involved in the increase in CYP4A.

### Direct correlation between CYP4A expression and steatosis phenotypes

**Fig. 3:**
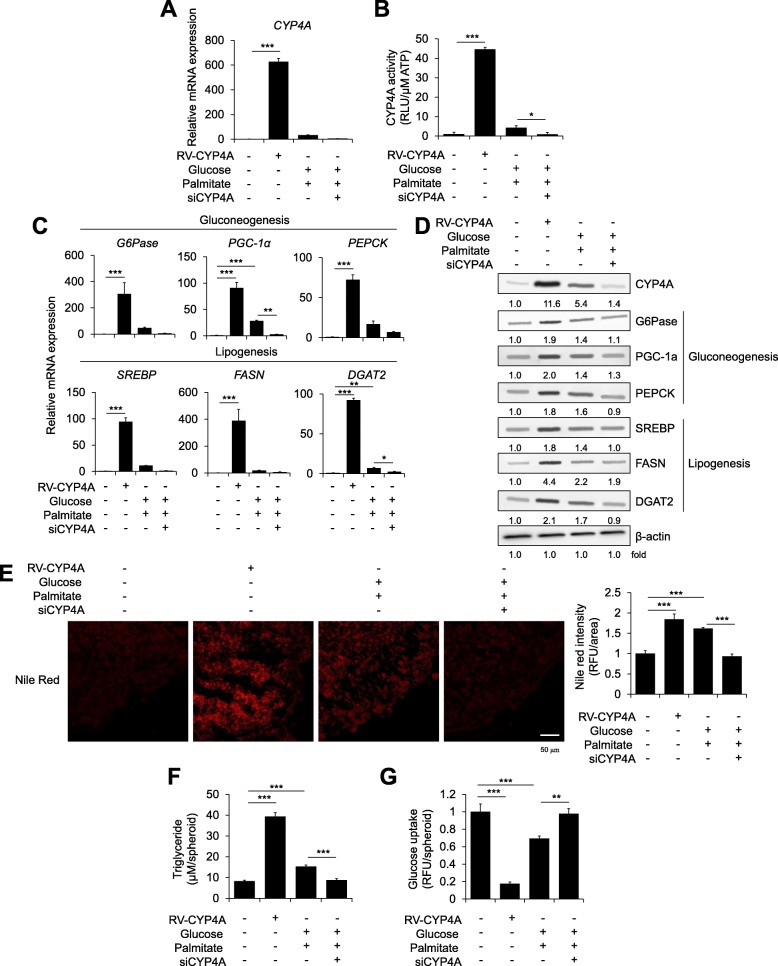
Changes in the steatosis phenotype upon CYP4A overexpression or knockdown in a novel 3D hepatic steatosis model. HepaRG cells were transduced with retroviral CYP4A (RV-CYP4A) or transfected with an siRNA for CYP4A (siCYP4A) and then 3D cultured with HUVECs and MSCs for 10 days. Steatosis was induced with glucose/palmitate for the last 5 days. **a** mRNA expression levels of CYP4A and **b** CYP4A activity were determined in each indicated 3D spheroid; *n* = 3. **c** mRNA expression levels of gluconeogenesis- and lipogenesis-related genes were determined in each 3D spheroid; *n* = 3. β-Actin was used as an internal control. **d** Protein expression levels of CYP4A and gluconeogenesis- and lipogenesis-related genes were determined in each group. **e** Intracellular lipids were stained with Nile red (left) and quantified by fluorescence intensity (right); *n* = 3. **f** The triglyceride concentration was quantified by absorbance in each treated 3D spheroid; *n* = 3. **g** Glucose uptake was quantified by fluorescence intensity; *n* = 3. *p < 0.05, **p < 0.01, and ***p < 0.001

To identify the direct effects of CYP4A on steatosis pathology, we over-expressed CYP4A by introducing the retroviral CYP4A gene or knocked down the gene by transfection with short interfering RNAs (siRNAs) in hepatic spheroids (Fig. 3a and b). CYP4A overexpression remarkably increased the expression of gluconeogenesis markers and lipogenesis markers compared with the levels in steatosis-induced spheroids, while CYP4A knockdown reduced the expression of these markers even under high-glucose- and palmitate-treated steatosis conditions (Fig. 3c and d). More importantly, CYP4A overexpression itself clearly enhanced neutral lipid droplet staining by Nile red (Fig. 3e) and triglyceride concentrations (Fig. 3f) without glucose and palmitate treatment, whereas CYP4A knockdown mitigated these steatosis phenotypes. Glucose uptake was also reduced in the CYP4A-overexpressing hepatic spheroids but not in CYP4A-knockdown steatosis-induced spheroids (Fig. 3g). Therefore, these results demonstrated that CYP4A expression directly correlated with steatosis pathology in our novel 3D hepatic steatosis model.

### Inhibition of CYP4A ameliorates steatosis phenotypes in a 3D hepatic steatosis model

**Fig. 4:**
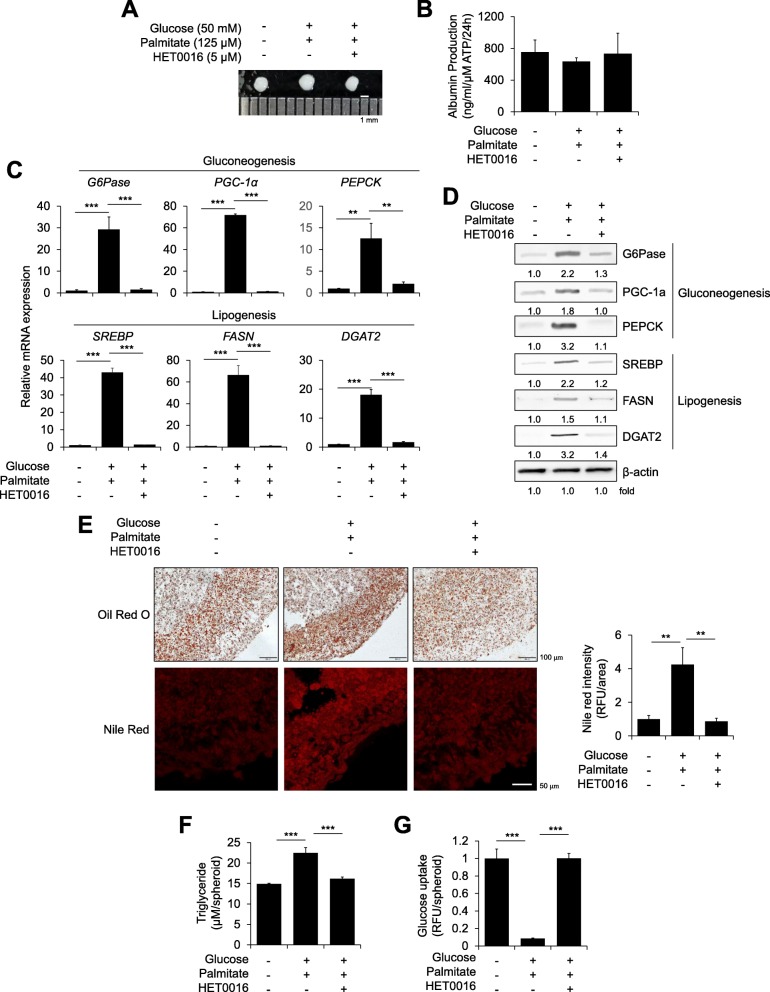
Effects of the CYP4A inhibitor HET0016 in a novel 3D hepatic steatosis model. **a** Gross morphology of control spheroids, glucose/palmitate-treated steatosis spheroids, and HET0016-treated steatosis spheroids. **b** ALB production was determined in each indicated spheroid normalized to ATP concentration; *n* = 3. **c** mRNA and **d** Protein expression levels of gluconeogenesis- and lipogenesis-related genes were determined in each indicated spheroid; *n* = 3. β-Actin was used as an internal control for mRNA and protein expression. **e** Intracellular lipids were stained with Oil red O (top) and Nile red (bottom) and quantified by Nile red fluorescence intensity (right); *n* = 3. **f** The triglyceride concentration was quantified by absorbance in each indicated spheroid; *n* = 3. **g** Glucose uptake was quantified by fluorescence intensity. **p < 0.01 and ***p < 0.001

Next, to determine whether targeting CYP4A could attenuate hepatic steatosis, we administered HET0016, a selective inhibitor of CYP4A [22], to high-glucose- and palmitate-induced steatosis spheroids. Preliminary, results confirmed the inhibitory effect of HET0016 on CYP4A activity in HepG2 cells, a human hepatoma cell line (Additional file 1: Figure S3A). HET0016 decreased lipid droplet intensity (Additional file 1: Figure S3B) and restored glucose uptake (Additional file 1: Figure S3C) in 2D-cultured palmitate-treated HepG2 cells. Morphologically, steatosis-induced and HET0016-treated spheroids were indistinguishable (Fig. 4a). Albumin production was slightly decreased in steatosis-induced spheroids but not in HET0016-treated steatosis spheroids (Fig. 4b). HET0016 treatment decreased the mRNA expression of gluconeogenesis and lipogenesis markers by up to 10- to 55-fold compared with that in steatosis-induced spheroids (Fig. 4c). Protein levels were also decreased by HET0016 treatment in steatosis-induced spheroids (Fig. 4d). The increased intensity of Oil red O or Nile red also appeared to decrease after HET0016 treatment in steatosis-induced spheroids (Fig. 4e). Compared with that in untreated steatosis-induced spheroids, the triglyceride concentration was decreased (Fig. 4f), and glucose uptake was recovered (Fig. 4g) by HET0016 treatment. These data revealed that inhibition of CYP4A could alleviate steatosis phenotypes in our novel 3D hepatic steatosis model.

### Inhibition of CYP4A reduces ER stress and partially recovers insulin signaling in a novel 3D hepatic steatosis model

**Fig. 5:**
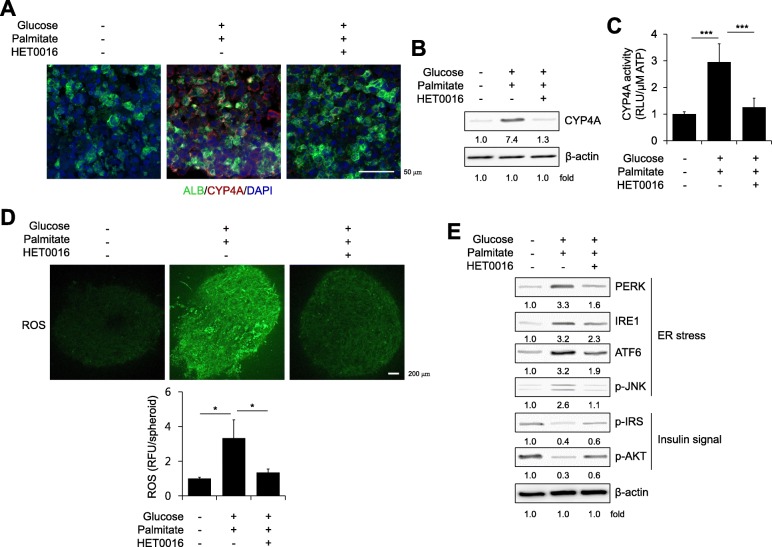
Inhibition of CYP4A reduces ER stress and partially recovers insulin signaling in a novel 3D hepatic steatosis model. **a** ALB and CYP4A expression was determined within cryosections of control spheroids, glucose/palmitate-treated steatosis spheroids, and HET0016-treated steatosis spheroids. The nuclei were counter-stained with DAPI (blue). **b** Protein expression levels and **c** Activity of CYP4A were determined in each indicated spheroid; *n* = 3. **d** Each indicated spheroid was stained with H2DCFDA (top) and quantified by fluorescence intensity (bottom); *n* = 3. **e** Expression levels of ER stress markers and insulin signal components were determined by Western blot analysis in each indicated spheroid. β-Actin was used as an internal control. *p < 0.05 and ***p < 0.001

Previous reports by our group and others have demonstrated that CYP4A is involved in the generation of reactive oxygen species (ROS), which are responsible for lipid peroxidation and subsequently cause ER stress and insulin resistance [2, 21, 31, 32]. Palmitate-induced ROS generation (Additional file 1: Figure S4A) and ER stress-induced ROS generation [induced by tunicamycin (Additional file 1: Figure S4B) or thapsigargin (Additional file 1: Figure S4C)] were clearly reduced by treatment with the CYP4A inhibitor HET0016 in 2D-cultured HepG2 cells. The increased expression (Fig. 5a and b) and activity (Fig. 5c) of CYP4A and the ROS generation (Fig. 5d) in steatosis spheroids were distinctly decreased by HET0016 treatment, which contributed to a decrease in ER stress signaling, such as PERK, IRE1, ATF6, and p-JNK, and partially recovered insulin signaling, such as p-insulin receptor substrate 1 (p-IRS) and p-AKT (Fig. 5e). Overall, these results demonstrate that targeting CYP4A by HET0016 treatment attenuates hepatic steatosis by ameliorating ER stress and improving insulin signaling in our novel multicellular organotypic liver model.

## Discussion

Despite years of intense research effort, effective therapeutics targeting hepatic steatosis to halt disease progression at an early stage are still limited. The lack of reliable in vitro human liver model systems is a critical hurdle for drug discovery studies. Previously, we found that CYP4A influenced diabetic fatty liver development by inducing hepatic ER stress, insulin resistance, and apoptosis in mouse models [21]. Here, we confirmed the value of CYP4A in our novel human cell-based multicellular organotypic liver model that included 1) HepaRG cells 2) in triple co-culture with HUVECs and MSCs, 3) self-organization on a soft matrix, and 4) an improved generation efficiency of 3D spheroids using conical tubes; we describe this method here for the first time (Fig. 1a).

HepaRG cells are progenitors with a high proliferation ability and the capacity to differentiation into hepatocytes with high CYP activity [11, 33, 34]. Triple co-culture itself with non-parenchymal cells such as HUVECs and MSCs improved the expression of hepatic-specific markers, even in 2D culture (Fig. 1c), and self-organization in 3D culture on a soft matrix substantially increased ALB production (Fig. 1d) and CYP3A4 activity (Fig. 1e) consistent with previous reports [35, 36]. Biomechanical alterations induced by soft niches with supporting cells may provide a more physiologically relevant model, and we previously reported that the use of a soft matrix to mimic the dynamic liver microenvironment facilitated HepaRG function in 3D culture [29]. Our present Matrigel layer (mixed 1:1 with medium, approximately 4.0 mg/ml final concentration) provides a stiffness of approximately 0.02 kPa or less, as it has been reported that the stiffness of 4.4 mg/ml of Matrigel is approximately 0.02 kPa [37], reflecting the soft microenvironment of the liver. Here, we further developed a pathologically relevant model using high glucose and the long-chain free fatty acid, palmitate, which are known to increase lipid accumulation in vivo and are mainly derived from adipose tissue and a HFD [38]. This milieu increased the expression of gluconeogenesis, lipogenesis, ER stress, and insulin resistance markers within 5 days in our model (Figs. 2, 3, and 5); these phenotypic alterations are largely observed in metabolic syndromes such as T2DM and NAFLD [38]. Thus, we provide here for the first time a HepaRG-based multicellular 3D hepatic steatosis model.

CYPs are a superfamily of proteins that contain heme as a co-factor and are essential for the metabolism and detoxification of endogenous and exogenous substances [39, 40]. Specifically, the omega-hydroxylase CYP4A is mostly expressed in the liver and kidney and is responsible for the metabolism of fatty acids and prostaglandin [41]. Increased CYP4A expression was observed in the liver of *db*/*db* mice, ob/ob mice, and HFD- or methionine and choline-deficient (MCD) diet-induced mouse models in reports from our group and others [21, 23–25]. Forced CYP4A expression in the liver of wild-type mice resulted in severe hepatic lipid accumulation, while *CYP4a14−/−* mice were resistant to HFD- and MCD-induced hepatic steatosis and fibrosis [25]. Knockdown of *CYP 4a* in the livers of *db*/*db* mice using shRNA or inhibition of CYP4A by treatment with the selective inhibitor HET0016 [22, 42] ameliorated glucose tolerance, insulin sensitivity, and ER stress [21]. These aforementioned in vivo hepatic phenotypes associated with *CYP4a* expression are consistent with our in vitro data observed in a novel 3D hepatic steatosis model (Figs. 4 and 5).

CYP4A is known as a potential hepatic source of ROS, which leads to lipid peroxidation and subsequently promotes ER stress and inflammatory responses [43]. Human livers with NAFLD have increased oxidative cellular damage in association with clinico-pathological features [44]. We also found that increased ROS production induced by free fatty acids was reduced by treatment with HET0016, a selective inhibitor of CYP4A (Fig. 5d), and inhibition of CYP4A by HET0016 reverted dysregulated lipid metabolism (Fig. 4), resulting in reduced ER stress signaling (Fig. 5e) in our 3D hepatic steatosis model. Overall, our novel multicellular organotypic liver model accurately recapitulates pathophysiological phenotypes associated with liver diseases and could serve as a translational platform for modelling human responses.

## Conclusions

We provide here for the first time a HepaRG cell-based multicellular 3D hepatic steatosis model. Self-organization of HepaRG cells, HUVECs, and MSCs in triple co-culture on a soft matrix using a conical tube readily improved the generation efficiency of 3D spheroids and further promoted functional competence. More importantly, simulating hepatic steatosis-related pathologies by treatment with excess glucose and palmitate strongly recapitulated NAFLD phenotypes correlated with CYP4A, and targeting CYP4A with a specific inhibitor or siRNA clearly alleviated features of hepatic steatosis. Therefore, our novel multicellular 3D hepatic model can serve as an alternative and advanced human cell-based in vitro biomimetic liver system for translational medicine approaches targeting various hepatic diseases.

## Methods

### Cell culture

Undifferentiated HepaRG cells (HPR101, Biopredic International, Saint Grégoire, France) were cultured in William’s E medium (Thermo Fisher Scientific, Waltham, MA, USA), supplemented with 1X Glutamax (Thermo Fisher Scientific), 10% fetal bovine serum (FBS, FBS-BBT-5XM, RMBIO, Missoula, MT, USA), 50 U/ml penicillin and 50 μg/ml streptomycin (1% PS) (Thermo Fisher Scientific), 1X Insulin-Transferrin-Selenium (ITS) (Thermo Fisher Scientific), and 5 × 10^− 5^ M hydrocortisone hemisuccinate (Santa Cruz Biotechnology, Dallas, TX, USA). The medium was changed every 2 days, and the cells were passaged at a 1:4 ratio after 12 days of culture. HUVECs were isolated from human umbilical cords with approval from the Public Institutional Bioethics Committee designated by the MOHW (IRB File No. P01–201712–31-004). Human umbilical cord (hUC) was washed with 70% EtOH and phosphate-buffered saline (PBS), and a 3-way stopcock was inserted into the hUC vein and tied. To remove blood, 20 ml of RPMI1640 (Thermo Fisher Scientific) was injected through the cock and squeezed out from the cord. One end of the hUC was gripped by a hemostatic clamp, 20 ml of collagenase type IV (210 units/ml, Thermo Fisher Scientific) was injected, and the hUC was then incubated for 30 min at 37 °C. After incubation, collagenase solution was collected into a 50-ml conical tube, 20 ml of RPMI1640 supplemented with 10% FBS was injected, and the solution was re-collected into the previous 50-ml conical tube. The collected cells were centrifuged at 1,000 rpm for 7 min, and the cell pellets were resuspended in HUVEC culture medium containing Medium 199 (Thermo Fisher Scientific) supplemented with 20% FBS, 10 ng/ml basic fibroblast growth factor (bFGF, PeproTech, Rocky Hill, NJ, USA), 5 units/ml heparin (Sigma-Aldrich, St. Louis, MO, USA), and 1% PS on a 0.2% gelatin-coated plate. The medium was changed every 2 days. Human umbilical cord blood (hUCB) MSCs were purchased from CEFO Bio (Seoul, Korea) and maintained in Minimum Essential Medium Eagle, Alpha Modification (MEM-α, Thermo Fisher Scientific) supplemented with 10% FBS, 1X MEM non-essential amino acids (Thermo Fisher Scientific), 1X Glutamax I, and 1% PS. The medium was changed every 2 days. The HepG2 (ATCC, Manassas, VA, USA) human liver cancer cell line was cultured in Dulbecco’s modified Eagle’s medium (Thermo Fisher Scientific) supplemented with 10% FBS and 1% PS. The medium was changed every 2 days.

### Hepatic spheroid formation

Matrigel™ (Corning incorporated, Corning, NY, USA) was diluted with HUVEC medium (200 μl:200 μl) in a 15 ml conical tube and solidified in a 37 °C incubator for at least 30 min. Undifferentiated HepaRG cells (1.6 × 10^6^ cells/spheroid), HUVECs (2 × 10^5^ cells/spheroid), and hUCB MSCs (2 × 10^5^ cells/spheroid) were resuspended in a mixture of HepaRG and HUVEC culture medium (4:1, v:v) and applied to the pre-solidified Matrigel bed. On day 1, the medium was replaced with fresh medium containing 1% dimethyl sulfoxide (DMSO, Sigma-Aldrich) for adaptation. On day 2, the medium was replaced with fresh medium containing 1.7% DMSO for hepatic differentiation. The medium containing 1.7% DMSO was changed every 2 days throughout the culture period.

### Immunocytochemistry

Hepatic spheroids were fixed with 4% paraformaldehyde for 15 min at room temperature (RT), immersed in 30% sucrose, and embedded in OCT compound (Sakura Finetek USA Inc., Torrance, CA, USA). Frozen sections were permeabilized with 0.1% Triton X-100 and blocked with 4% bovine serum albumin (Sigma-Aldrich) for 2 h at RT. The samples were stained with the respective primary antibody diluted in blocking buffer overnight at 4 °C, washed with 0.05% Tween-20 (Sigma-Aldrich) in PBS and then incubated with Alexa Fluor-conjugated secondary antibodies (Thermo Fisher Scientific) for 1 h at RT. Florescence images were captured with an Olympus microscope (Olympus, Tokyo, Japan). The antibodies used are listed in Additional file 2: Table S1.

### Real-time polymerase chain reaction (PCR)

Total RNA was prepared from samples using a TRIzol Kit according to the manufacturer’s instructions (Thermo Fisher Scientific). Reverse transcription was performed with Topscript RT DryMIX following the manufacturer’s instructions (Enzynomics, Daejeon, Korea). Quantitative real-time PCR was performed by a 7500 Fast Real-Time PCR System (Applied Biosystems, Waltham, MA, USA) using Fast SYBR Green Master Mix (Applied Biosystems). The primer sequences used in this study are presented in Additional file 3: Table S2.

### ALB production

To measure ALB production, 100 μl of culture medium was collected from each group and quantified using an enzyme-linked immunosorbent assay kit (Bethyl Laboratories, Montgomery, TX, USA) according to the manufacturer’s protocol. The total ALB concentration was normalized to the total adenosine triphosphate (ATP) content of each sample using a CellTiter-Glo 3D cell viability assay kit (Promega, Madison, WI, USA).

### CYP3A4 and CYP4A activity

CYP3A4 and CYP4A activity was analysed using a P450-Glo assay kit with Luciferin-IPA for CYP3A4 and Luciferin-4A for CYP4A according to the manufacturer’s protocol (Promega). Luciferase activity was measured using a Victor X Light luminometer (PerkinElmer, Waltham, MA, USA), and the data were normalized based on the ATP content obtained by a CellTiter-Glo 3D cell viability assay kit.

### Generation of hepatic steatosis spheroid and staining of lipid droplets

To generate hepatic steatosis spheroids, treatment with 50 mM glucose (Thermo Fisher Scientific) and 125 μM palmitate (Sigma-Aldrich) was started on day 7 of self-organization and continued for 5 days. For Oil red O staining, frozen sections were incubated with 60% isopropanol for 5 min at RT, the Oil red O solution (Sigma-Aldrich) was immediately changed, and the sections were incubated for 30 min at RT. Oil red O solution was removed, and the sections were washed with distilled water (DW) more than 5 times. Lipid accumulation was observed with a light microscope (Olympus). For Nile red staining, frozen sections were stained with 10 μg/ml Nile red (Thermo Fisher Scientific) solution for 5 min at RT in the dark. The Nile red solution was removed, and the sections were washed 3 times with PBS. Lipid accumulation was observed with a fluorescence microscope. The intensity of Nile red staining was measured using ImageJ.

### Western blot analysis

Hepatic spheroids were lysed with RIPA buffer (Thermo Fisher Scientific) containing a protease inhibitor cocktail (Merck Millipore, Frankfurter, Germany) on ice for 20 min and centrifuged at 20,000 x g for 30 min at 4 °C. Protein concentrations were determined using a Bradford protein assay kit (Bio-Rad, Hercules, CA, USA). Total protein (10 μg) was separated via Mini-PROTEAN TGX Gels (Bio-Rad) and electrotransferred to PVDF membranes (Bio-Rad) using a Trans-Blot Turbo Transfer System (Bio-Rad). The membranes were blocked in 5% skim milk (BD Biosciences) at RT for 2 h and then incubated with a specific primary antibody overnight at 4 °C. The membranes were washed with PBS containing 0.1% Tween-20 and then probed with HRP-conjugated secondary antibodies (Santa Cruz Biotechnology). The bands were detected using SuperSignal West Femto Chemiluminescent Substrate (Thermo Fisher Scientific) and LAS-3000 (Fujifilm, Minato, Tokyo, Japan). The bands were analysed using Image Gauge software (Fujifilm). The antibodies used in these experiments are listed in Additional file 2: Table S1.

### Triglyceride concentration

Hepatic spheroids were washed with cold PBS, resuspended and homogenized in 1 ml of 5% NP-40 solution (Sigma-Aldrich). The samples were slowly heated at 80–100 °C for 5 min and cooled to RT repeatedly to solubilize all triglycerides. The samples were centrifuged for 2 min at top speed to remove any insoluble materials and then diluted 10-fold with DW. The triglyceride concentration was analysed using a Triglyceride Assay Kit-Quantification (Abcam, Cambridge, MA, USA) following the manufacturer’s protocol, and absorbance was measured at 570 nm using a SpectraMax M3 microplate reader (Molecular Devices, San Jose, CA, USA).

### Glucose uptake

Glucose uptake was analysed using a Glucose Uptake Cell-Based Assay Kit (Cayman, Ann Arbor, MI, USA) according to the manufacturer’s instructions. Spheroids were transferred to a 96-well plate, and 200 μg/ml 2-NBDG, a fluorescent glucose analogue, was added to each spheroid and incubated for 1 h at 37 °C. The plate was centrifuged at 400 g for 5 min, the supernatant was aspirated, and 200 μl of Cell-Based Assay Buffer was added to each well. Centrifugation was repeated, 100 μl of Cell-Based Assay Buffer was added to each well, and fluorescence was detected using a microplate reader.

### Virus production

GP2–293 cells (Clontech, Mountain View, CA, USA) were transfected with pMX vectors containing CYP4A (Origene, Rockville, MD, USA) and the VSV-G envelope gene using Lipofectamine 2000 transfection reagent (Thermo Fisher Scientific). The supernatants were collected at 2–5 days after transfection and filtered using a 0.45-μm pore size filter (Merck Millipore, Burlington, MA, USA). The viruses were concentrated using an ultracentrifuge (Beckman Coulter, Brea, CA, USA) at 25,000 rpm (rotor: SW32Ti) for 120 min. Titers were quantified by counting green fluorescent protein-transduced cells using an Incucyte live-cell analysis system (Essen BioScience, Ann Arbor, MI, USA).

### Overexpression and knockdown experiments and HET0016 treatment

For overexpression experiments, HepaRG cells were pre-incubated for 1 h with 8 μg/ml polybrene (Sigma-Aldrich) and then transduced with concentrated CYP4A retroviruses at a multiplicity of infection of 3. For the knockdown experiment, HepaRG cells were transfected with siRNAs for CYP4A11 and CYP4A22 (Bioneer, Daejeon, Korea) using Dharmafect I transfection reagent (Dharmacon, Lafayette, CO, USA) according to the manufacturer’s instructions. For the HET0016 study, cells were pre-treated with 5 μM HET0016 (Cayman, Ann Arbor, MI, USA) for 6 h before replacing the culture medium with 50 mM glucose and 125 μM palmitate, and the treatment was continued for 5 days.

### ROS production

HepG2 cells (4 × 10^3^ cells per well for palmitate treatment, 1 × 10^4^ cells per well for tunicamycin or thapsigargin treatment) were seeded in a black clear-bottomed 96-well culture plate (Corning). After incubation overnight, the cells were pre-incubated with HET0016 for 6 h before palmitate (0.25 mM), tunicamycin (5 μg/ml) or thapsigargin (1 μM) stimulation. After 72 h (palmitate) or 24 h (tunicamycin or thapsigargin), the cell culture medium was replaced by PBS with 5 μM cell-permeant 2′,7′-dichlorodihydrofluorescein diacetate (H2DCFDA) (ThermoFisher Scientific). ROS production was measured by using a SpectroMax M4 (Molecular Devices). Representative figures were obtained from an Opera QEHS microscope with a 20X water lens (PerkinElmer).

### Statistical analysis

The data are representative of at least three independent biological replicates. The graphs present the mean ± SD of triplicate samples for CYP (CYP3A4 and CYP4A) activity, ALB production, PCR analysis, Nile red staining, glucose uptake, and triglyceride concentrations. The unpaired t-test (Fig. 1d and e, Additional file 1: Figure S1C, and S2) and one-way ANOVA followed by Bonferroni’s multiple comparisons test (Figs. 1c, 2b-c, e-g, and i, 3a-c and e-g, 4b-c and e-g, 5c and d, Additional file 1: Figure S1D-E, S3, and S4) were performed using GraphPad Prism version 5.00 for Windows (GraphPad Software, Inc., San Diego, CA, USA). A value of *p* < 0.05 was considered statistically significant.

## Additional files

Additional file 1: **Figure S1.** Optimization of hepatic spheroid formation. (A) Schematic diagram of our novel method for hepatic spheroid generation. HepaRG cells, HUVECs, and MSCs were co-assembled on a thick layer of Matrigel. Upper middle images present the well-organized 3D spheroids, and lower middle images present the failure of 3D cell clusters in a 24-well plate. The right image shows the representative gross morphology and an illustration of self-assembled hepatic spheroids on a Matrigel bed in a conical tube. (B) Schematic diagram of hepatic spheroid generation protocols. (C-E) mRNA expression levels of the indicated hepatocyte-specific markers were determined at different (C) time points during self-organization, (D) cell numbers, and (E) differentiation schedules using real-time PCR. β-Actin expression was used as an internal control; *n* = 3. *p < 0.05, **p < 0.01, and ***p < 0.001. **Figure S2.** Optimization of hepatic steatosis induction in 2D-cultured HepG2 cells. (A) Each indicated glucose and palmitate concentration was used to treat HepG2 cells for 7 days. Intracellular lipids were stained with Nile red (top) and quantified by red fluorescence intensity (bottom); *n* = 3. (B) Cell viability was measured by a cell counting kit-8 (CCK-8) assay in each indicated condition; *n* = 3. (C) mRNA expression levels of *SREBP* and *FASN* were determined in each indicated condition. β-Actin was used as an internal control; *n* = 3. *p < 0.05, **p < 0.01, and ***p < 0.001. **Figure S3.** Effects of the CYP4A inhibitor HET0016 in 2D-cultured HepG2 cells. (A) Activity of CYP4A was determined in retroviral CYP4A-transduced and 5 μM HET0016-treated HepG2 cells; *n* = 3. (B) Intracellular lipid droplets were stained with Nile red (left) and quantified by red fluorescence intensity (right) in each indicated condition; *n* = 3. (C) Glucose uptake was assayed (left) and quantified by fluorescence intensity (right) in each group; *n* = 3. *p < 0.05, **p < 0.01, and ***p < 0.001. **Figure S4.** Effects of the CYP4A inhibitor HET0016 on ROS generation induced by ER stress in 2D-cultured HepG2 cells. HepG2 cells treated with (A) palmitate (250 μM), (B) tunicamycin (5 μg/μl), or (C) thapsigargin (1 μM) for 72 hours were stained with H2DCFDA (left) and quantified by fluorescence intensity (right) in each indicated condition; *n* = 3. *p < 0.05, **p < 0.01, and ***p < 0.001. **Figure S5.** The extended data for each Western blot analysis. (PDF 1860 kb)

Additional file 2: **Table S1.** List of antibodies used in this study. (DOCX 15 kb)

Additional file 3: **Table S2.** List of primers used in this study. (DOCX 14 kb)

## Data Availability

All data generated or analyzed during this study are included in this published article and its additional files.

## Abbreviations

3D: Three-dimensional
ATF6: Activating transcription factor 6
CYP4A: Cytochrome P450 4A
DW: Distilled water
FBS: Fetal bovine serum
HFD: High-fat diet
hUC: Human umbilical cord
hUCB: Human umbilical cord blood
HUVECs: Human umbilical vein endothelial cells
IRE1: Inositol-requiring enzyme 1
MCD: Methionine and choline-deficient
MSCs: Mesenchymal stem cells
NAFLD: Non-alcoholic fatty liver disease
NASH: Non-alcoholic steatohepatitis
PBS: Phosphate-buffered saline
PERK: Protein kinase double-stranded RNA-dependent-like ER kinase
PHHs: Primary human hepatocytes
p-IRS: P-insulin receptor substrate 1
p-JNK: Phospho- c-jun N-terminal kinase
PS: Penicillin and streptomycin
RFU: Relative fluorescence unit
RLU: Relative luminescence unit
RT: Room temperature
T2DM: Type 2 diabetes mellitus
