# Supplementary material for: Targeting CYP4A attenuates hepatic steatosis in a novel multicellular organotypic liver model
Source: J Biol Eng. 2019 Aug 8;13:69. doi: 10.1186/s13036-019-0198-8 (PMC6686528; doi:10.1186/s13036-019-0198-8)

Figure S1

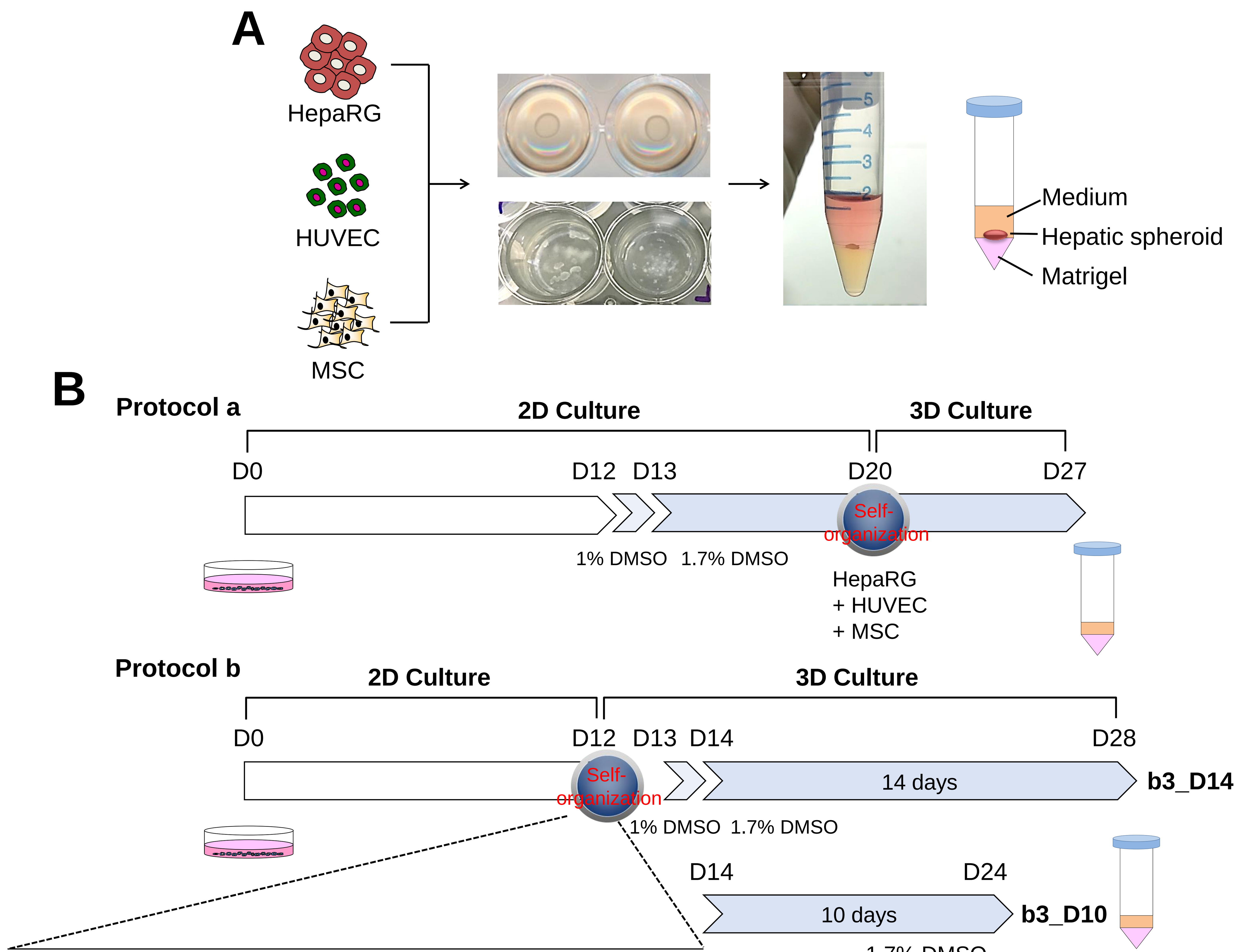

		HepaRG	HUVEC	MSC
**Protocol b1**	Cell No.	1 x 10^6	7 x 10^5	2 x 10^5
	Ratio	5	: 3.5	: 1
**Protocol b2**	Cell No.	1.4 x 10^6	4 x 10^5	2 x 10^5
	Ratio	7	: 2	: 1
**Protocol b3**	Cell No.	1.6 x 10^6	2 x 10^5	2 x 10^5
	Ratio	8	: 1	:1

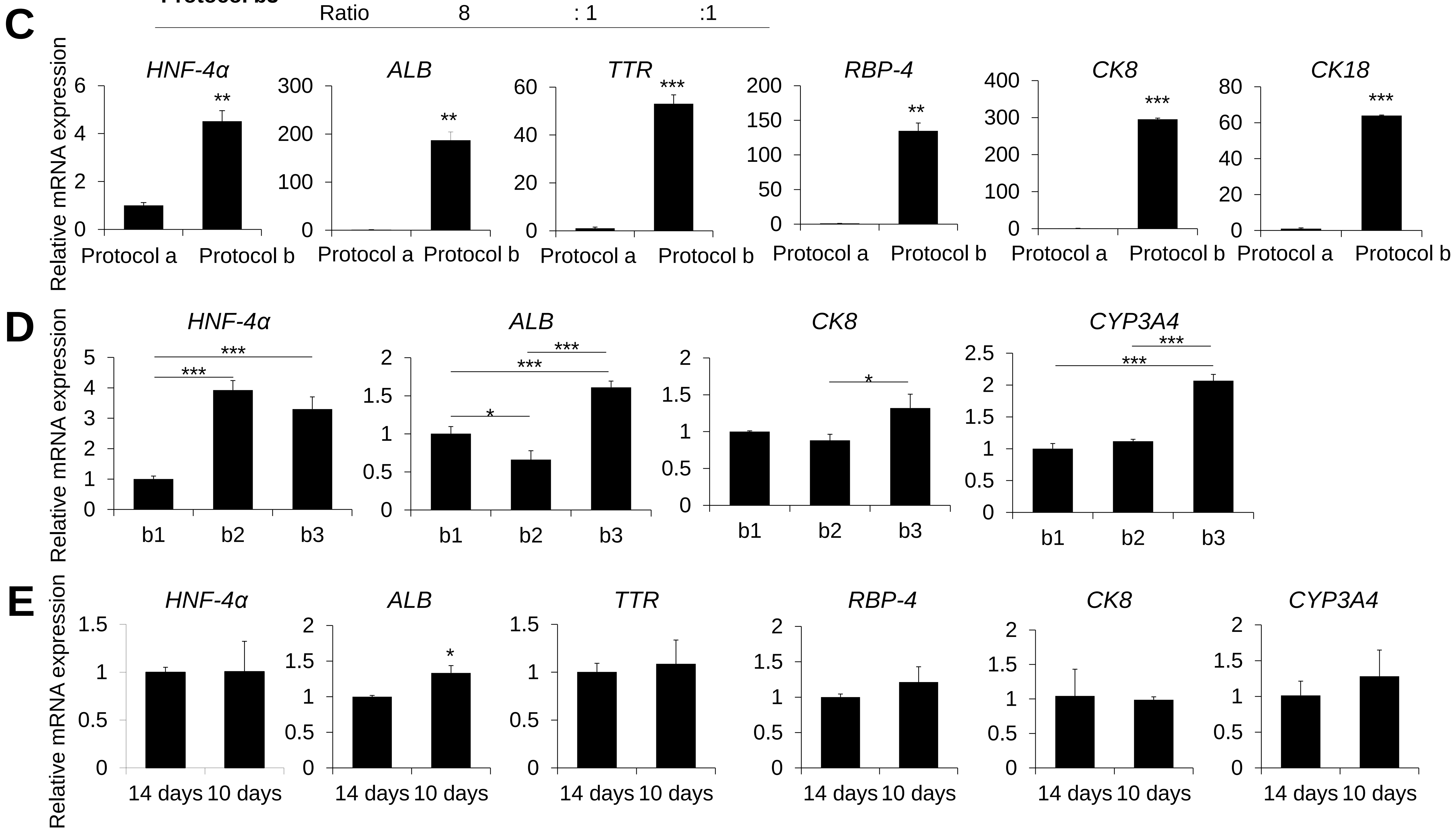

Figure S2

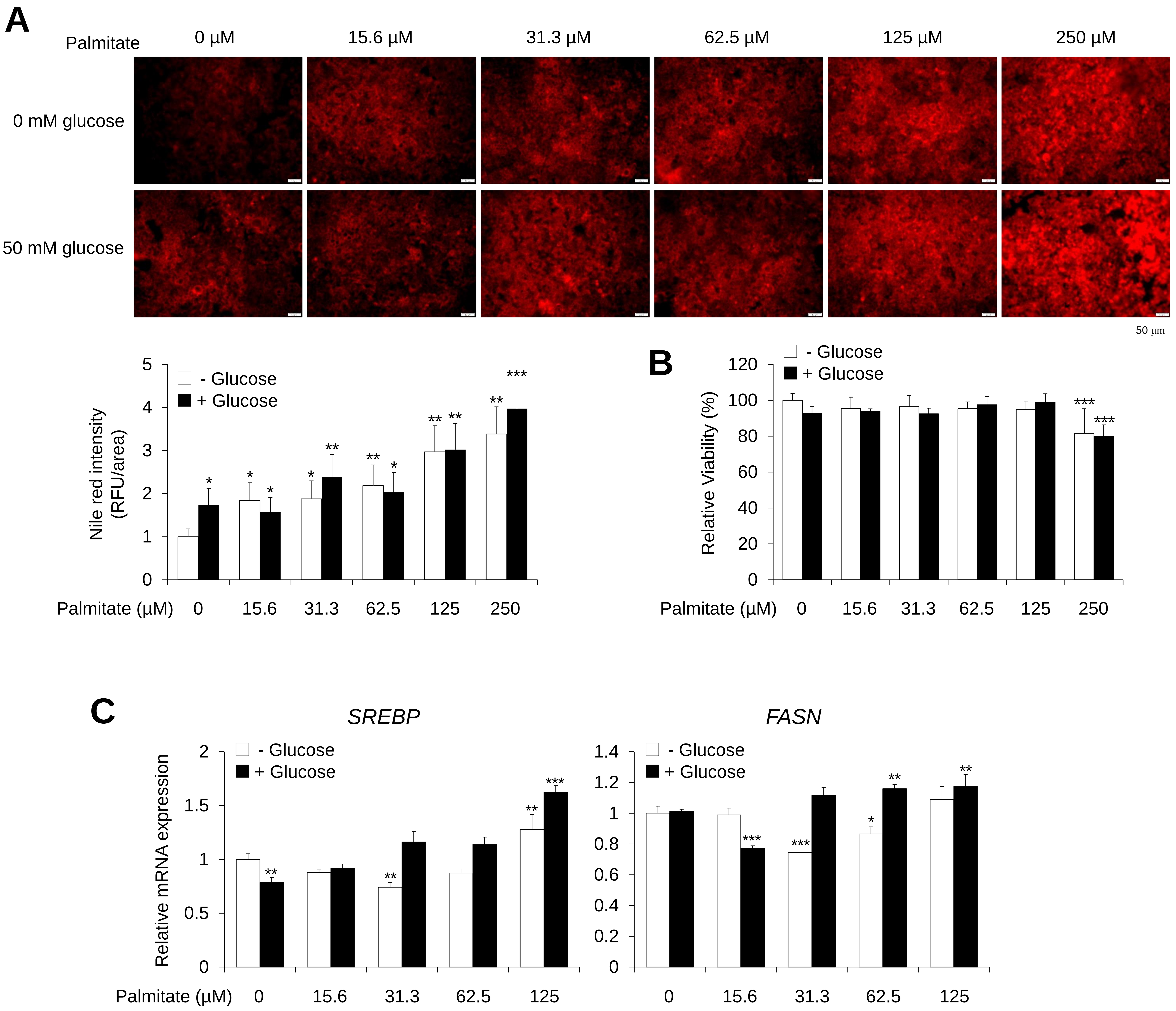

A
Palmitate
0 µM
15.6 µM
31.3 µM
62.5 µM
125 µM
250 µM
0 mM glucose
50 mM glucose
50 µm
- Glucose
+ Glucose
Nile red intensity (RFU/area)
Palmitate (µM)
0
15.6
31.3
62.5
125
250
B
Relative Viability (%)
C
SREBP
FASN
Relative mRNA expression

Figure S3

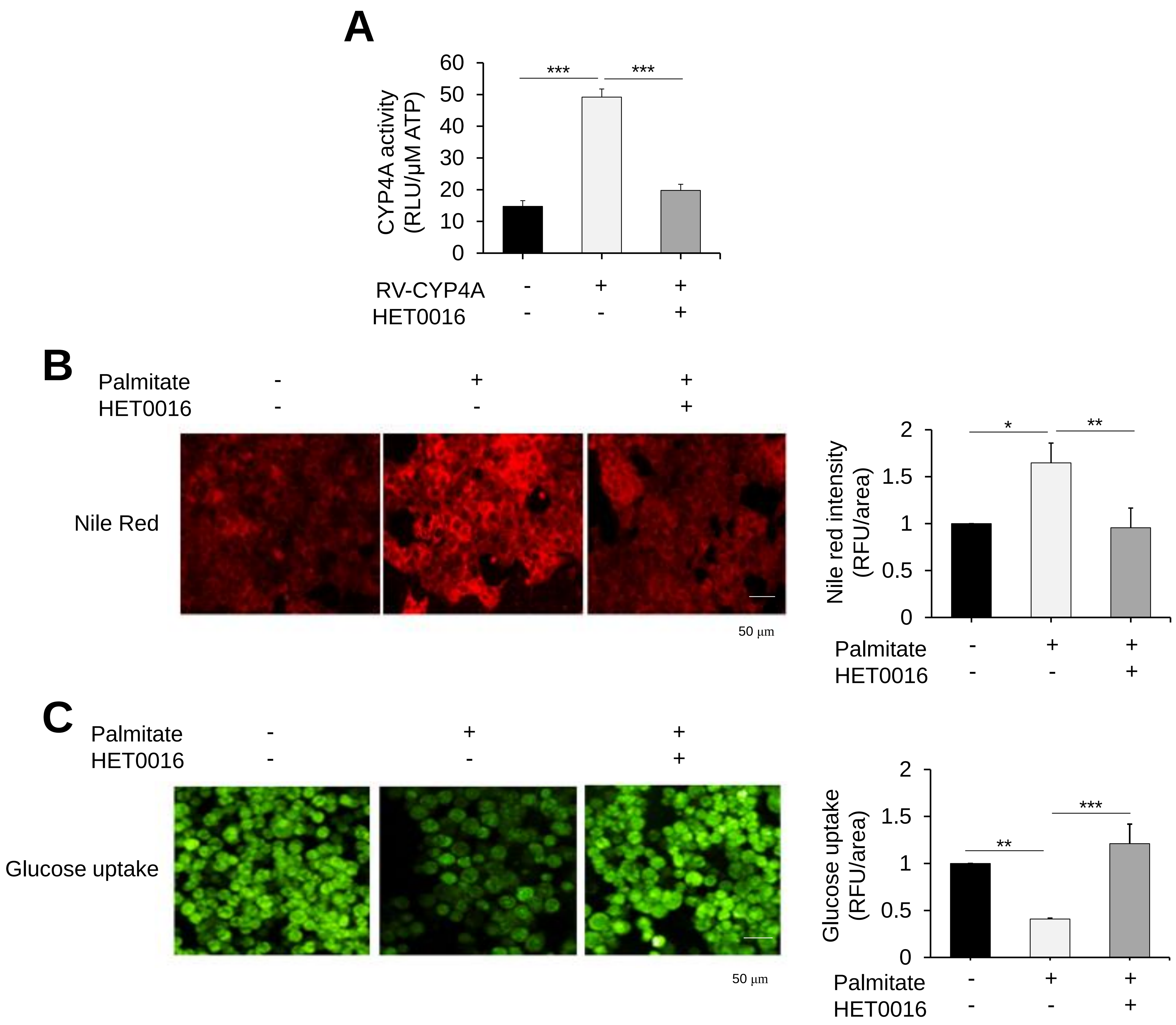
A
CYP4A activity
(RLU/µM ATP)
60
50
40
30
20
10
0
***
***
RV-CYP4A
-
+
+
HET0016
-
-
+
B
Palmitate
-
+
+
HET0016
-
-
+
Nile Red
50 µm
Nile red intensity
(RFU/area)
2
1.5
1
0.5
0
*
**
Palmitate
-
+
+
HET0016
-
-
+
C
Palmitate
-
+
+
HET0016
-
-
+
Glucose uptake
50 µm
Glucose uptake
(RFU/area)
2
1.5
1
0.5
0
**
***
Palmitate
-
+
+
HET0016
-
-
+

Figure S4

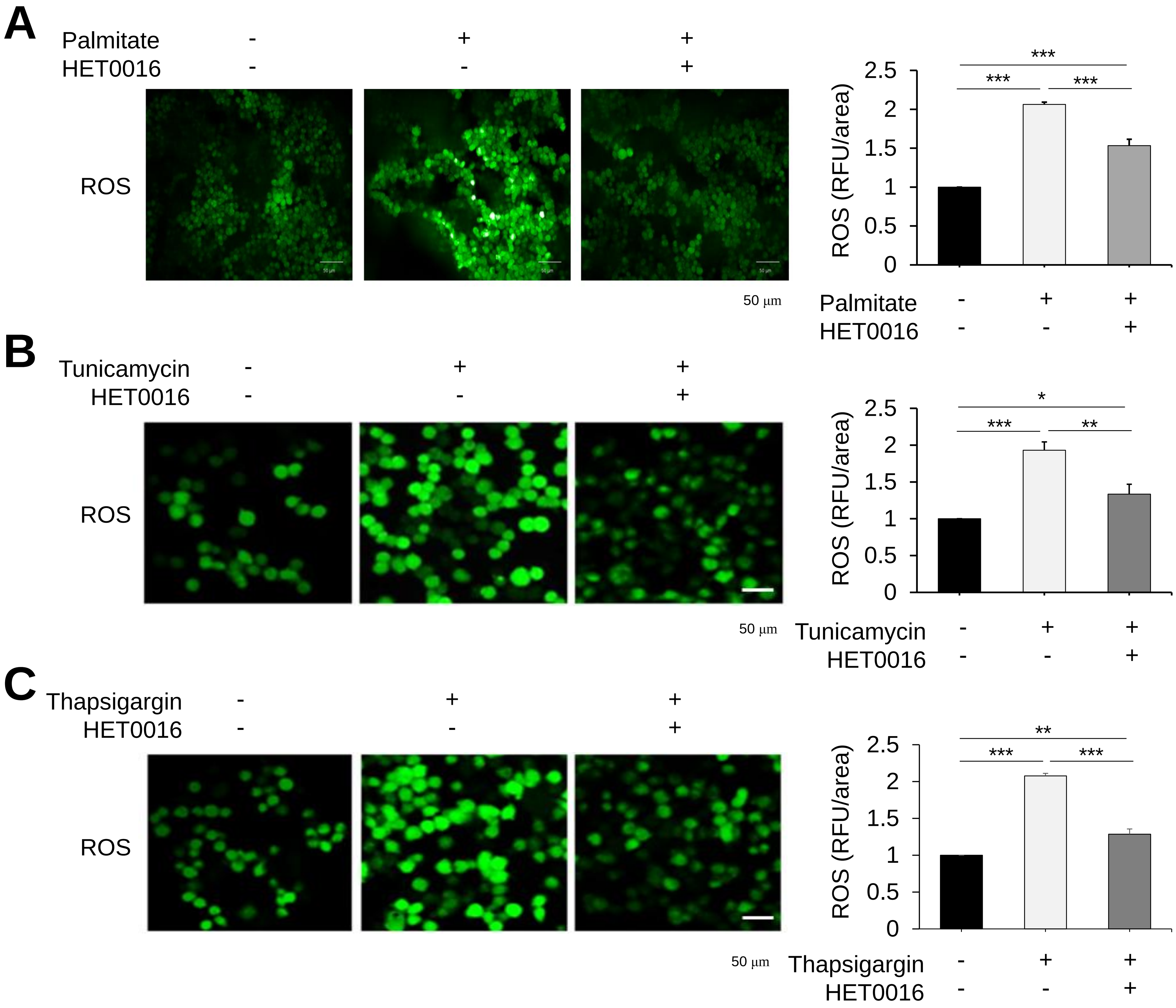

A
Palmitate - + +
HET0016 - - +
ROS
50 µm
ROS (RFU/area)
2.5
2
1.5
1
0.5
0
***
***
***
Palmitate - + +
HET0016 - - +
B
Tunicamycin - + +
HET0016 - - +
ROS
50 µm
ROS (RFU/area)
*
***
**
Tunicamycin - + +
HET0016 - - +
C
Thapsigargin - + +
HET0016 - - +
ROS
50 µm
ROS (RFU/area)
**
***
***
Thapsigargin - + +
HET0016 - - +

Figure S5 (figure continued on next page)

for Fig. 2D

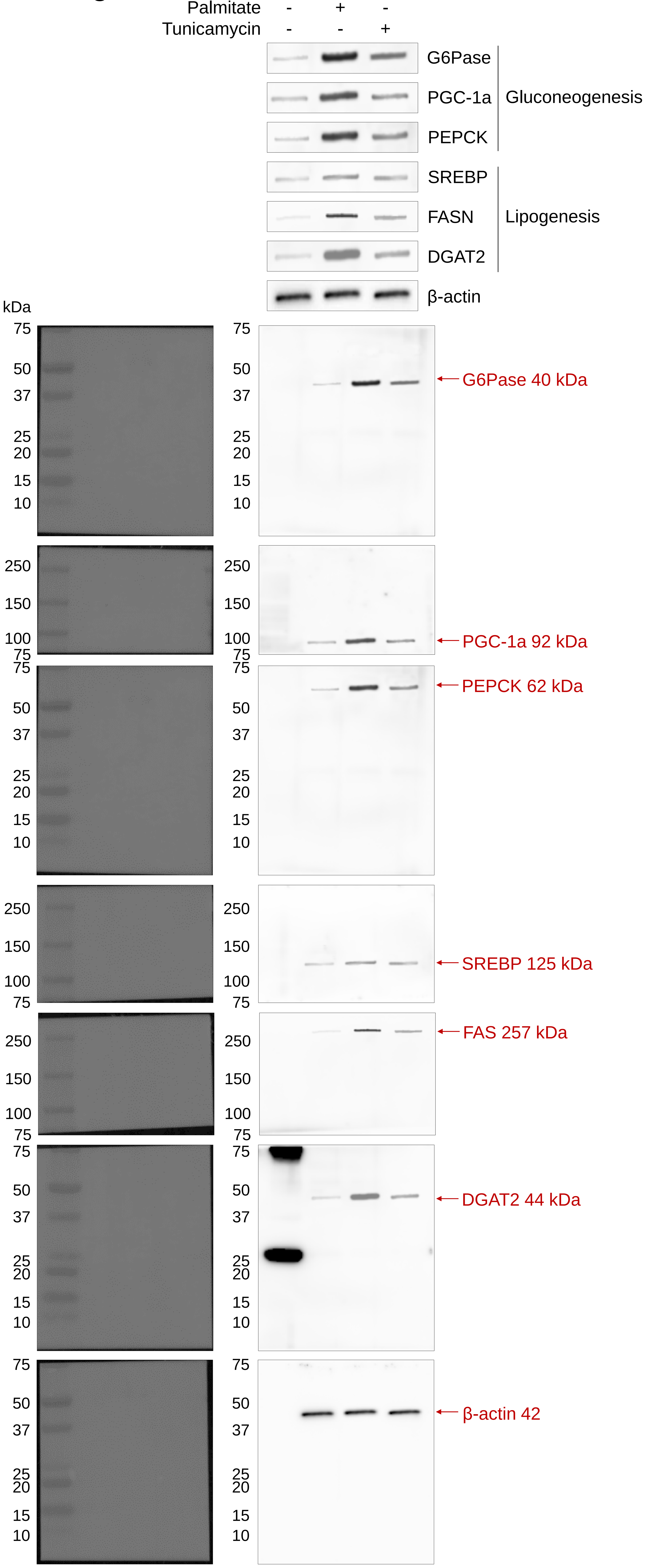

for Fig. 2H

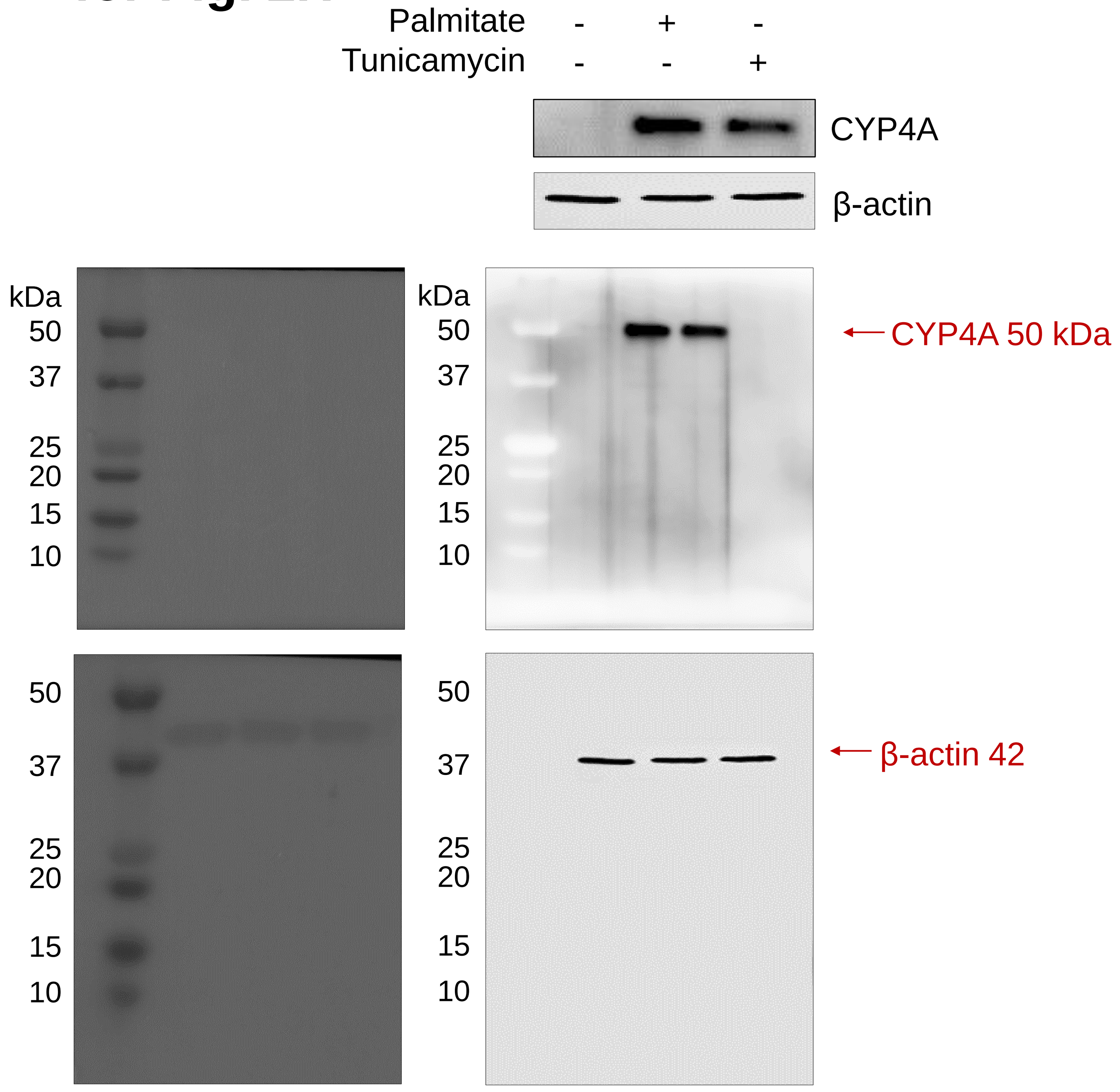

for Fig. 2J

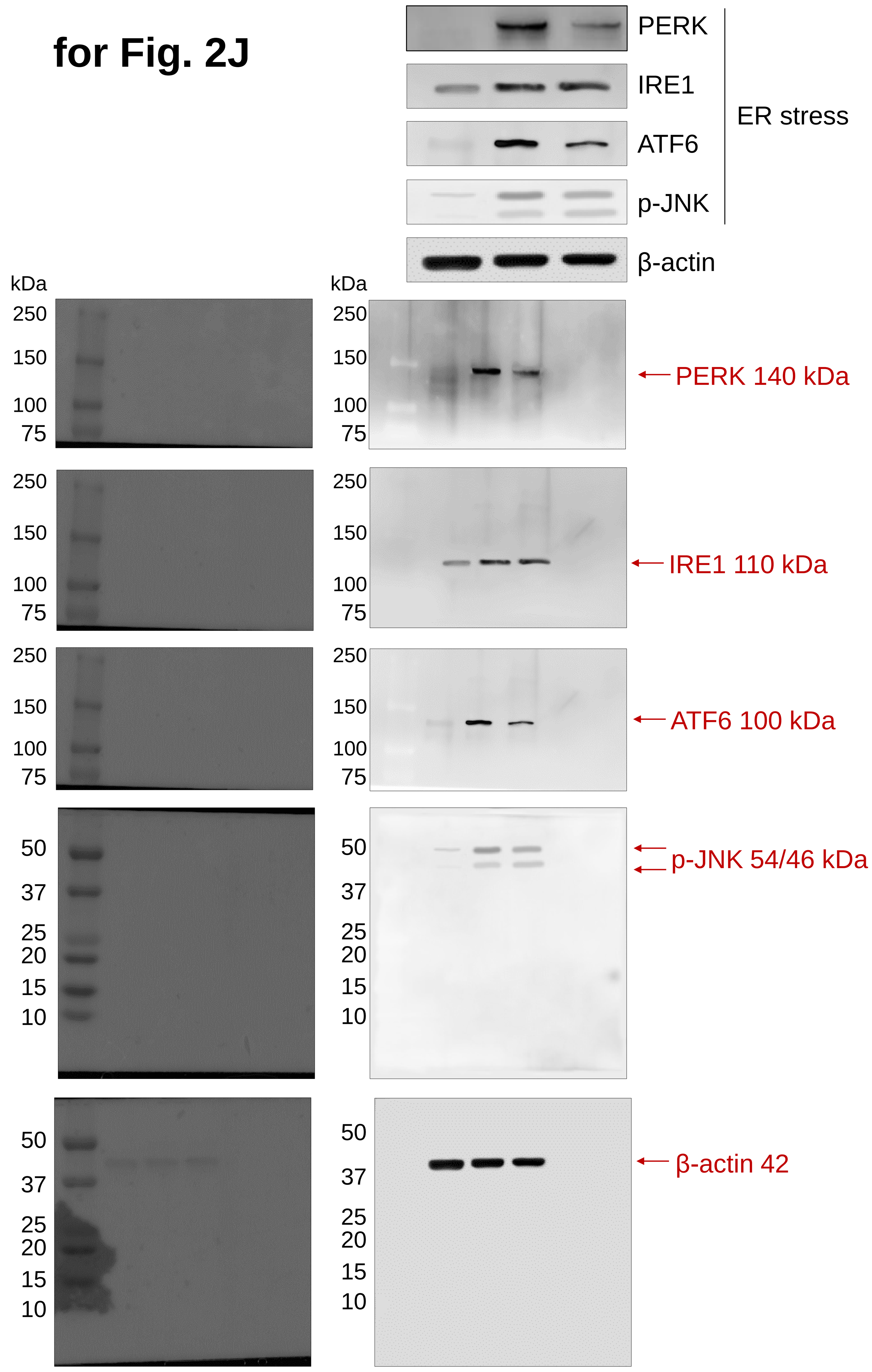

Figure S5 (figure continued on next page)

for Fig. 3D

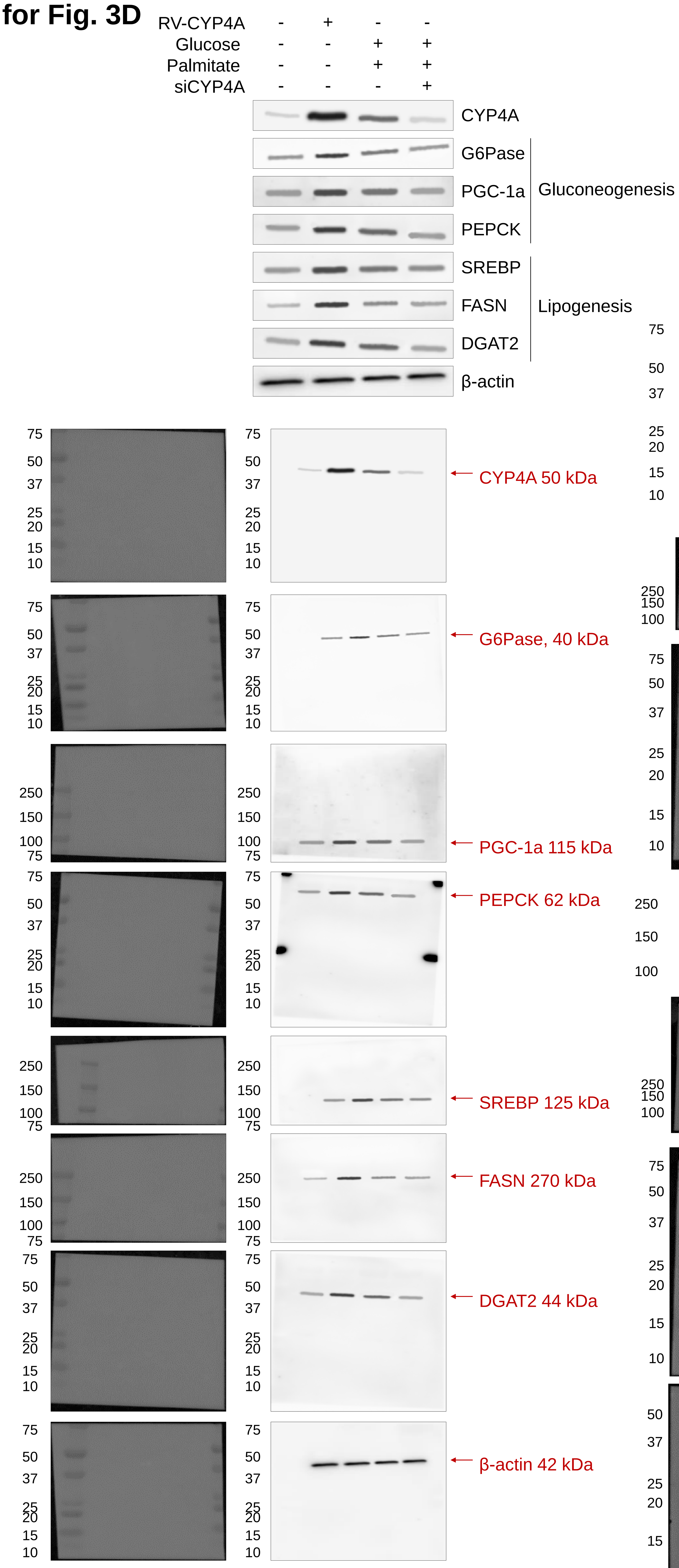

for Fig. 4D

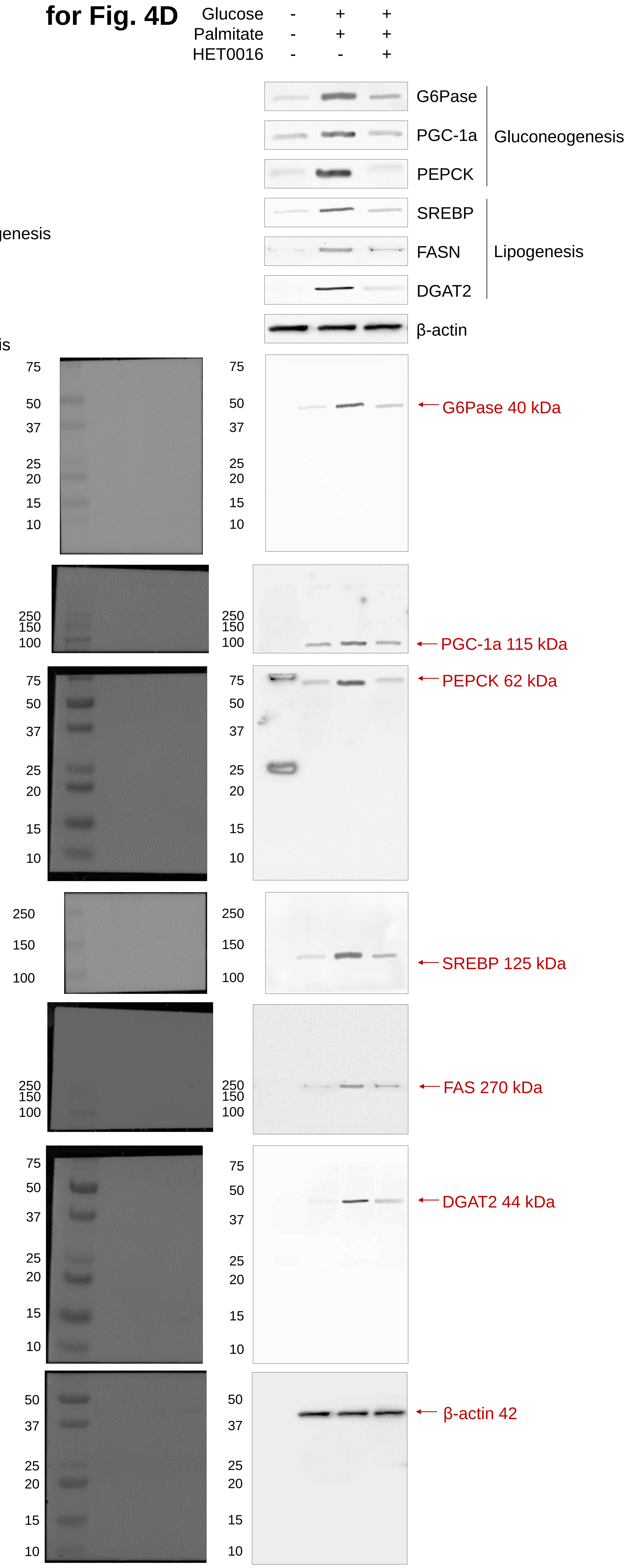

Figure S5

for Fig. 5B

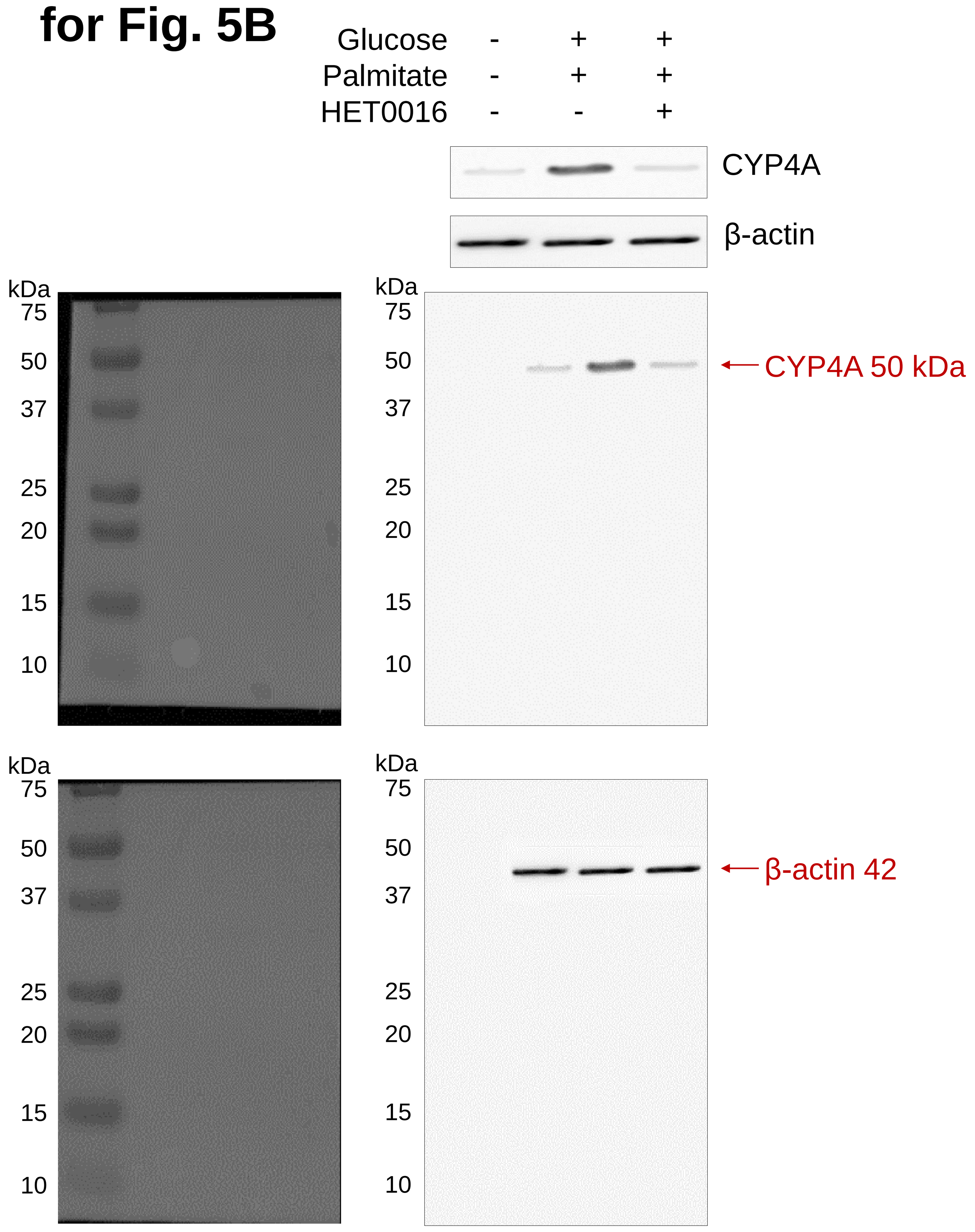

for Fig. 5E

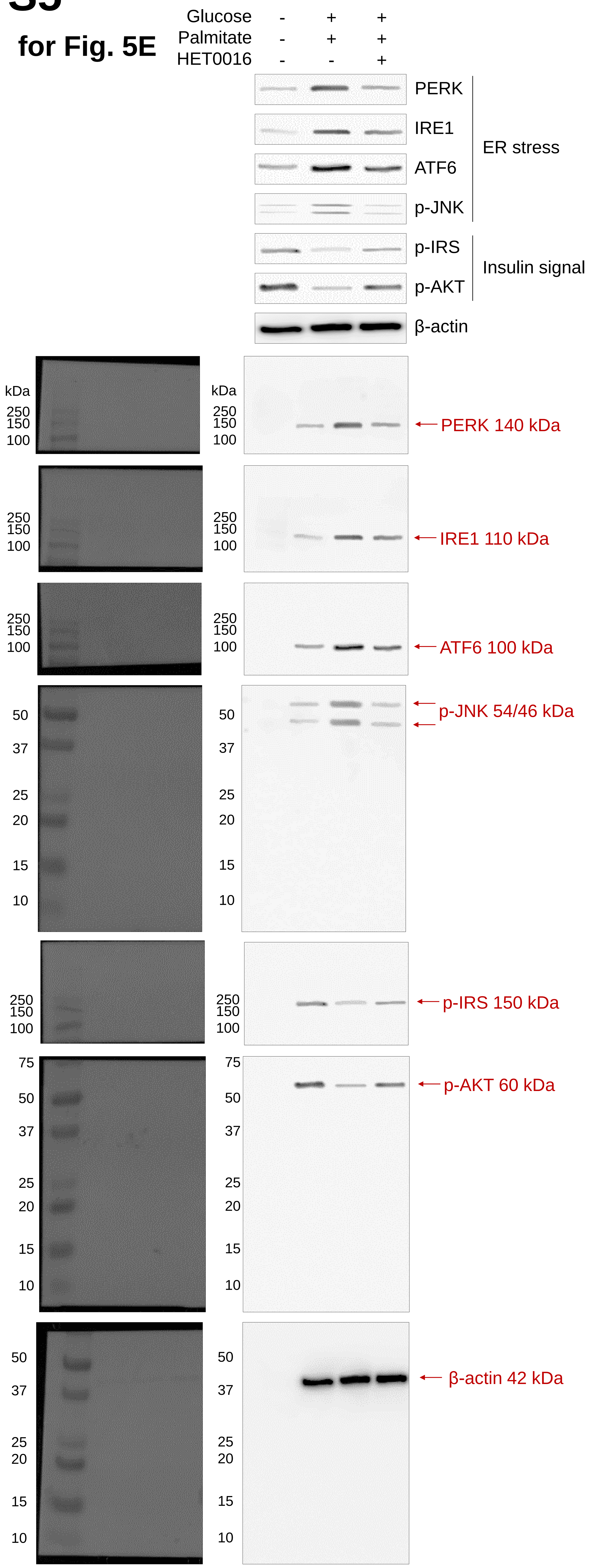

Supplement: Supplementary file 1 — Figure S1. Optimization of hepatic spheroid formation. (A) Schematic diagram of our novel method for hepatic spheroid generation. HepaRG cells, HUVECs, and MSCs were co-assembled on a thick layer of Matrigel. Upper middle images present the well-organized 3D spheroids, and lower middle images present the failure of 3D cell clusters in a 24-well plate. The right image shows the representative gross morphology and an illustration of self-assembled hepatic spheroids on a Matrigel bed in a conical tube. (B) Schematic diagram of hepatic spheroid generation protocols. (C-E) mRNA expression levels of the indicated hepatocyte-specific markers were determined at different (C) time points during self-organization, (D) cell numbers, and (E) differentiation schedules using real-time PCR. β-Actin expression was used as an internal control; n = 3. *p < 0.05, **p < 0.01, and ***p < 0.001. Figure S2. Optimization of hepatic steatosis induction in 2D-cultured HepG2 cells. (A) Each indicated glucose and palmitate concentration was used to treat HepG2 cells for 7 days. Intracellular lipids were stained with Nile red (top) and quantified by red fluorescence intensity (bottom); n = 3. (B) Cell viability was measured by a cell counting kit-8 (CCK-8) assay in each indicated condition; n = 3. (C) mRNA expression levels of SREBP and FASN were determined in each indicated condition. β-Actin was used as an internal control; n = 3. *p < 0.05, **p < 0.01, and ***p < 0.001. Figure S3. Effects of the CYP4A inhibitor HET0016 in 2D-cultured HepG2 cells. (A) Activity of CYP4A was determined in retroviral CYP4A-transduced and 5 μM HET0016-treated HepG2 cells; n = 3. (B) Intracellular lipid droplets were stained with Nile red (left) and quantified by red fluorescence intensity (right) in each indicated condition; n = 3. (C) Glucose uptake was assayed (left) and quantified by fluorescence intensity (right) in each group; n = 3. *p < 0.05, **p < 0.01, and ***p < 0.001. Figure S4. Effects of the CYP4A in [file 13036_2019_198_MOESM1_ESM.pdf]
