# Supplementary material for: Targeting CYP4A attenuates hepatic steatosis in a novel multicellular organotypic liver model
Source: J Biol Eng. 2019 Aug 8;13:69. doi: 10.1186/s13036-019-0198-8 (PMC6686528; doi:10.1186/s13036-019-0198-8)
Supplement: Supplementary file 2 — Table S1. List of antibodies used in this study. (DOCX 15 kb) [file 13036_2019_198_MOESM2_ESM.docx]

**Additional file 2: Table S1.** List of antibodies used in this study

| **Antibodies** | **Company** | **Catalog No.** | **Dilution** |
| --- | --- | --- | --- |
| anti-Albumin | A80-129A | Bethyl Laboratories | 1:200 (for IHC) |
| anti-ATF6 | NBP1-40256 | Novus Biologicals | 1:200 (for WB) |
| anti-β-actin | A1978 | Sigma-Aldrich | 1:500,000 (for WB) |
| anti-CD31 | ab9498 | Abcam | 1:30 (for IHC) |
| anti-CYP4A | ab140635 | Abcam | 1:100 (for IHC)  1:1,000 (for WB) |
| Anti-Desmin | AB907 | Millipore | 1:50 (for IHC) |
| anti-DGAT2 | sc-293211 | Santa Cruz Biotechnology | 1:200 (for WB) |
| anti-FAS | sc-48357 | Santa Cruz Biotechnology | 1:1,000 (for WB) |
| anti-G6Pase | ab83690 | Abcam | 1:100 (for WB) |
| anti-IRE-1 | ab37073 | Abcam | 1:500 (for WB) |
| anti-PEPCK | sc-271029 | Santa Cruz Biotechnology | 1:100 (for WB) |
| anti-PERK | #9451 | Cell Signaling Technology | 1:1,000 (for WB) |
| anti-PGC-1a | sc-13067 | Santa Cruz Biotechnology | 1:200 (for WB) |
| anti-phospho-AKT | #9271 | Cell Signaling Technology | 1:1,000 (for WB) |
| anti-phospho-IRS-1 | #3667 | Cell Signaling Technology | 1:1,000 (for WB) |
| anti-phospho-JNK | #5599 | Cell Signaling Technology | 1:1,000 (for WB) |
| anti-SREBP | sc-365513 | Santa Cruz Biotechnology | 1:200 (for WB) |
| anti-Mouse-HRP | sc-2005 | Santa Cruz Biotechnology | 1:1,000 (for WB) |
| anti-Rabbit-HRP | sc-2004 | Santa Cruz Biotechnology | 1:1,000 (for WB) |
| anti-Goat-Alexa488 | A11055 | Thermo Fisher Scientific | 1:200 (for IHC) |
| anti-Mouse-Alexa488 | A11001 | Thermo Fisher Scientific | 1:200 (for IHC) |
| anti-Mouse-Alexa594 | A21125 | Thermo Fisher Scientific | 1:200 (for IHC) |
| anti-Rabbit-Alexa594 | A21442 | Thermo Fisher Scientific | 1:200 (for IHC) |
