# Supplementary material for: Targeting CYP4A attenuates hepatic steatosis in a novel multicellular organotypic liver model
Source: J Biol Eng. 2019 Aug 8;13:69. doi: 10.1186/s13036-019-0198-8 (PMC6686528; doi:10.1186/s13036-019-0198-8)
Supplement: Supplementary file 3 — Table S2. List of primers used in this study. (DOCX 14 kb) [file 13036_2019_198_MOESM3_ESM.docx]

**Additional file 3: Table S2.** List of primers used in this study.

| **Gene** | **Primer (Forward)** | **Primer (Reverse)** |
| --- | --- | --- |
| *ALB* | TTTATGCCCCGGAACTCCTTT | AGTCTCTGTTTGGCAGACGAA |
| *β-actin* | GGACTTCGAGCAAGAGATGG | AGCACTGTGTTGGCGTACAG |
| *CK8* | TCATAGACAAGGTACGGTTCC | GCCTAAGGTTGTTGATGTAGC |
| *CK18* | GAGCTGCTCCATCTGTAGGG | CACAGTCTGCTGAGGTTGGA |
| *CYP3A4* | CTTCATCCAATGGACTGCATAAAT | TCCCAAGTATAACACTCTACACAGACAA |
| *CYP4A* | CACCACAACCCAAAAGTGTG | GCAGTTCCTTGATCCTCCT |
| *DGAT2* | AGTGGCAATGCTATCATCAT | GAGGCCTCGACCATGGAAGAT |
| *FASN* | GTACACACCCAAGGCCAAGTA | GACGTGGACGGATACTTTCC |
| *G6pase* | TGGTTGGGATTCTGGGCTGT | TCTACACCCAGTCCCTTGAG |
| *HNF-4α* | GGCCAAGTACATCCCAGCTTT | CAGCACCAGCTCGTCAAGG |
| *PEPCK* | GTTCAATGCCAGGTTCCCAG | TTGCAGGCCAGTTGTTGAC |
| *PGC-1a* | TGCCCTGGATTGTTGACATGA | TTTGTCAGGCTGGGGGTAGG |
| *RBP-4* | GAGTTCTCCGTGGACGAGAC | TCCAGTGGTCATCATTTCCTTTC |
| *SREBP* | TCAGCGAGGCGGCTTTGGAGCAG | CATGTCTTCGATGTCGGTCAG |
| *TTR* | TGGGAGCCATTTGCCTCTG | AGCCGTGGTGGAATAGGAGTA |
